# Migraine headache: a review of the molecular genetics of a common disorder

**DOI:** 10.1007/s10194-012-0478-x

**Published:** 2012-09-01

**Authors:** Cherubino Di Lorenzo, Gaetano S. Grieco, Filippo M. Santorelli

**Affiliations:** 1 Don Carlo Gnocchi Onlus Foundation, Rome, Italy; 2 Laboratory of Neurogenetics, C. Mondino National Institute of Neurology Foundation, IRCCS, Pavia, Italy; 3 Molecular Medicine and Neurodegenerative Diseases-IRCCS Stella Maris, Pisa, Italy; 4 Child Neurology, University of Pisa, Pisa, Italy; 5 Molecular Medicine, IRCCS Stella Maris, via dei Giacinti 2, 56128 Calambrone, Pisa Italy

**Keywords:** Migraine, Genetics, Familial hemiplegic migraine (FHM), CADASIL, MELAS, Pharmacogenomics

## Abstract

This tutorial summarises the state-of-the-art on migraine genetics and looks at the possible future direction of this field of research. The view of migraine as a genetic disorder, initially based on epidemiological observations of transmission of the condition within families, was subsequently confirmed by the identification of monogenic forms of “syndromic” migraine, such as familial hemiplegic migraine. We are currently witnessing a change in the way genetic analysis is used in migraine research: rather than studying modalities of inheritance in non-monogenic forms of migraine and in the persistent modalities of migraine headache, researchers are now tending to focus on the search for genetic markers of dysfunction in biological systems. One example of the evolution of migraine genetic research is provided by the recent efforts to shed light on the pharmacogenomic mechanisms of drug response in migraineurs. In addition, novel molecular approaches about to be introduced are expected to further increase knowledge on this topic and improve patient management.

## Introduction

Migraine is a common disorder characterised by recurrent disabling attacks of headache associated with nausea, vomiting, hypersensitivity to light, sound, and smell (migraine without aura, MO), and, in about 25 % of cases, neurological aura symptoms (migraine with aura, MA) [1, 2]. Aura symptoms, which generally include visual disturbances, last for up to an hour but can sometimes last for several days. Rarely, focal motor seizures may occur as part of the aura spectrum [1]. Patients with at least one MA attack per month show a higher risk for brain lesions [3]. The neurobiological mechanism underlying migraine aura is cortical spreading depression [4].

Migraine is a major cause of non-fatal disease-related disability [5], being estimated to affect about 12 % of the Western population. The disease is more frequent in females (3:1 female-to-male ratio) and has its peak prevalence between the ages of 22 and 55 years [5, 6]. Overall, MA prevalence is 1–4 % in the male population and 3–10 % in the female population [6].

Onset of the disease may occur in childhood or even infancy, and according to international guidelines, more than 30 % of migraineurs are candidates for preventive therapy. In general, migraine has a profound effect on wellbeing and general functioning, not only during attacks but also in terms of work performance, family and social relationships, and school achievement [2].The WHO rates it among the most disabling, and costly, chronic disorders [7]. Nonetheless, it is generally estimated that about 30 % of affected individuals do not receive a correct diagnosis and are likely to remain inadequately treated, or even misdiagnosed throughout their lives [8]. Prophylactic drug treatment of migraine should be considered in a series of situations: when quality of life, work commitments, or school attendance are severely impaired; when the frequency of attacks per month is two or higher; when migraine attacks do not respond to acute drug treatment; when patients experience frequent, very long, or uncomfortable auras [2].

The common observation that migraine tends to run in families has long been the basis for suggesting that genetic determinants play a significant role in the disease. It has been shown that at least 50 % of migraineurs have a parent affected by a similar condition and a familial liability has been confirmed in several studies, especially ones comparing concordance rates between monozygotic and dizygotic twins (as recently reviewed [9]). Although familial does not necessarily mean genetic, epidemiological evidence seems to indicate a close gene–environment interaction, at least in MA. The possible role of brain energy through oxidative metabolism has also been invoked [10]. Deeper insight into the possible molecular genetic contribution to migraine has come from the study of rarer, monogenic forms of “syndromic” migraine, such as familial hemiplegic migraine (FHM), as well as from the use of technically improved molecular methods.

The aim of this paper is to briefly review current molecular genetic evidence in both MA and MO, focusing on recent data from whole-genome studies; we will also consider the outlook for research in this field, destined to be driven by constant advances in gene/genome sequencing technology. A useful technical “glossary” is shown in Table 1.

**Table 1:** Glossary of useful “technical” terminology

Genotype	It is the state of the pairs of alleles present at one or more loci associated with a given trait
Phenotype	It refers to the observable state of the trait (e.g. blue eyes, red hair)
Dominant	It refers to mutations at a given locus occurring in a heterozygote status
Recessive	It refers to mutations occurring in homozygosity
Loss of function	It refers to the functional consequences of mutations on protein function. It indicates that the amount of normal protein is decreased (as seen in inborn errors of metabolism)
Gain of function	It refers to the functional consequences of mutations on protein function. It indicates the case of abnormal gene dosage, as in trisomy of chromosome 21, or when mutations result in a negative effect on normal protein function)
Inappropriate expression	It indicates abnormal protein expression as can often be seen for oncogenes
Incomplete or no penetrance	It is the case of individuals who may only partly display the characteristic disease phenotype
Variable expression	It is the variable consequence of gene mutation on clinical phenotype. It is believed to be due to allelic/locus heterogeneity or to the effects of modifier genes (or even environmental or metabolic factors)
Incomplete dominance	It refers to the blending of traits that occurs when two different alleles of a gene pair occur together and neither is dominant
Co-dominance	It refers to the condition in which both the alleles in a gene pair are fully expressed, without one being dominant over the other (this results in a third, novel phenotype)
Polygenic inheritance	This non-Mendelian inheritance is determined by the alleles of more than one gene. The more genes involved, the greater the number of intermediate phenotypes that will be produced. This modality occurs with a sort of additive effect and the picture becomes even more variegated when the multiple genes interact with environmental factors
Cytoplasmic transmission	Another aspect of non-Mendelian inheritance that occurs in the case of variants in the mitochondrial genome (mtDNA). MtDNA is a circular, double-stranded, 16.569 base-pair molecule of DNA which encodes 13 essential polypeptides for the oxidative phosphorylation (OXPHOS) system, two ribosomal RNAs, and 22 tRNAs. The mitochondrial genome is strictly maternally inherited and there are several hundred to several thousands of copies within a single cell [10]. The number of copies present varies between different cell types, depending on the energy demand within the tissue, and it is extremely high in neurons [10]

## Monogenic forms of migraine: familial hemiplegic migraine

The most straightforward approach for identifying genes and unravelling genetic pathways involved in complex genetic disorders is to study monogenic subtypes of the same disorders. Familial hemiplegic migraine (FHM), a rare form of migraine with motor aura, is an example of a monogenic subtype of migraine which can be considered a model for the common forms of the disease, because, with the exception of the hemiparesis, it presents with exactly the same headache and aura features [11]. In FHM, the symptoms include both typical migraine attacks and severe episodes with prolonged aura and impaired consciousness, ranging from confusion to profound coma. In some cases, attacks can be triggered by minor head trauma [12], and in others, epilepsy may be a co-morbid condition or occur during a hemiplegic attack. In 20 % of families, patients also have fixed cerebellar symptoms and signs, such as nystagmus and progressive ataxia. A clearer link with common forms of MA and MO has emerged in patients without a proven or suspected family history (sporadic hemiplegic migraine, SHM). In a large Danish population-based study, an increased risk of MA and MO was demonstrated in first-degree relatives of SHM patients [13]. Although FHM and MA share many similarities on clinical grounds, it remains unclear whether and to what extent they are pathophysiologically related. To date, three genes—two ion-channel genes and one encoding an ATP exchanger—have been found to underlie FHM. *CACNA1A* (FHM1), located on chromosome 19p13 [14], was the first FHM gene identified and approximately 70 different causal missense mutations have been detected in it. Besides FHM, these mutations can be associated with episodic cerebellar ataxia type 2, cerebellar ataxia type 6 (SCA6) [15], and episodic seizures and migraine with motor regression [16]. *CACNA1A* encodes the α1 subunit of neuronal Ca_V_2.1 (P/Q-type) voltage-gated calcium channels that are widely expressed throughout the central nervous system [17]. This subunit is involved in voltage sensitivity and mutations lead to uptake of Ca^2+^ ions into neurons in response to a smaller depolarisation than is required by wild-type channels. This, in turn, causes excessive release of the neurotransmitter glutamate [18].

**Fig. 1:**
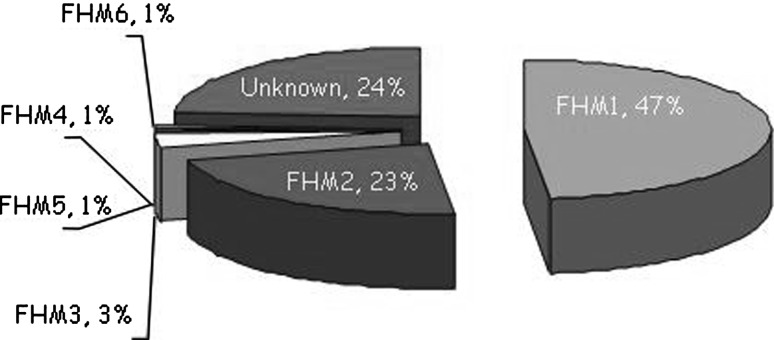
Relative frequency of known forms of familial hemiplegic migraine (FHM). See text for details

The second FHM gene, *ATP1A2* (FHM2), is located on chromosome 1q23 [19] and encodes the α2 subunit of sodium–potassium pumps. There are now over 40 FHM2 mutations that, with rare exceptions, are private, that is, they occur in single families. Most of the *ATP1A2* mutations are associated with pure FHM, without additional clinical symptoms [19–23]. However, FHM2 mutations are increasingly being recognised to be associated with cerebellar problems [24], childhood convulsions (benign familial infantile convulsions) [25], epilepsy [20, 26], alternating hemiplegia of childhood [27], and permanent mental retardation [20, 28]. Intriguingly, mutations in *ATP1A2* have also been shown to be associated with non-hemiplegic migraine phenotypes, such as basilar migraine [29] and even common migraine [30], although this causality has not been definitively confirmed. The most recently identified FHM gene is *SCN1A* (FHM3), located on chromosome 2q24 [31], which is the site of five known FHM mutations [32–34]. *SCN1A* encodes the α1 subunit of neuronal Na_V_1.1 voltage-gated sodium channels and is a well-known epilepsy gene harbouring over 100 truncating and missense mutations associated with childhood epilepsy (i.e. severe myoclonic epilepsy of infancy) [35, 36]. It is of note that two carriers of the FHM3 Q1489H mutation also suffered from ‘elicited repetitive transient daily blindness’ and childhood epilepsy [37]. There exist forms of typical FHM not related to mutations in FHM genes and further heterogeneity is expected to emerge. For instance, a yet unknown FHM4 gene on chromosome 14, and the possible FHM5/*SLC4A4* and FHM6/*SLC1A3* genes encoding metabolic transporters are believed to exist (Fig. 1) (reviewed in [38]) but even more loci/genes are anticipated.

There are no obvious clinical differences between carriers of mutations in the three genes already known to cause FHM, although patients with FHM1 mutations more often exhibit cerebellar ataxia and FHM2 cases report (minor) head trauma as a trigger of attacks. In addition, for all three FHM genes, there are mutation carriers who have epilepsy (or present with it). This is not particularly surprising given the epidemiological evidence of a bidirectional co-morbidity between migraine and epilepsy, which suggests that both disorders have, at least in part, a shared pathophysiology [39]. The identification of gene mutations that can cause both FHM and epilepsy would provide a unique opportunity to study these mechanisms.

## Functional studies of FHM mutations

The functional consequences of FHM gene mutations have been extensively analysed in cellular and animal models. Studies of heterologous expression of human mutations and calcium channel functioning using whole-cell electrophysiology (for review see [40]) have shown that FHM1 mutations increase the opening probabilities of channels, also at more negative voltages, compared to what is seen in wild-type channels [41, 42]. It is hypothesised that this gain-of-function effect results in increased Ca^2+^ influx, and therefore predicts increased neurotransmission. A similar conclusion was reached in knock-in mice harbouring the human FHM1 R192Q mutation. The threshold for CSD was found to be lowered and CSD propagation velocity increased in FHM1 R192Q mutant mice. These observations, highly relevant to migraine, indicate that FHM1 mutant mice are useful models for studying the pathophysiology of the disease in vivo. The functional consequences of a large number of *ATP1A2* mutations causing either FHM or SHM have been investigated in vitro. FHM2 mutations resulted in reduced or absent sodium potassium pump activity with decreased (in the case of T345A and A606T variants) or increased (in some other cases) affinity for potassium [43–45]. Thus, the consequence of FHM2 mutations is a loss-of-function mechanism with non-functional proteins impairing pump function. Unfortunately, it has not proved possible to reproduce these cell data in animal models that lack the α2-subunit, because Atp1a2 knockout mice have a severe phenotype and die immediately after birth because of their inability to start breathing [46]. The functional consequences of FHM3 mutations have been investigated both in heterologous systems [31, 32] and in cultured neurons [47], and both gain- and loss-of-function mechanisms have been observed.

**Fig. 2:**
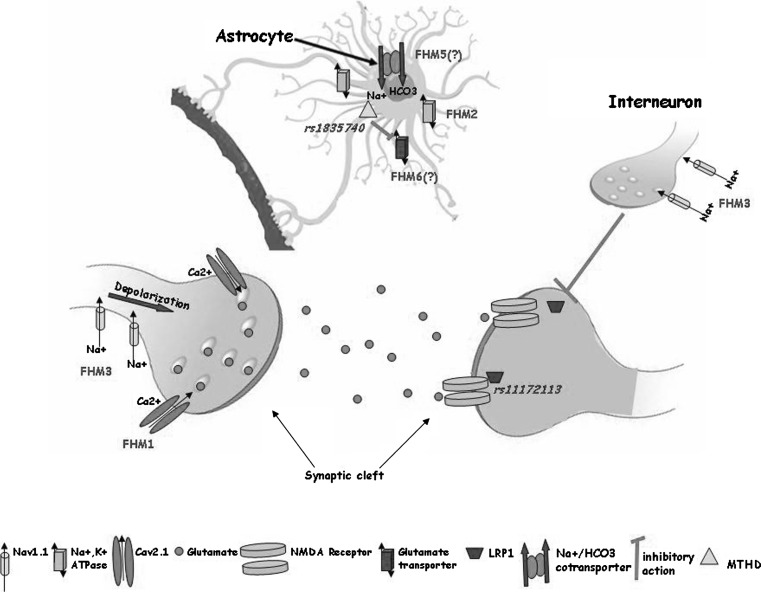
Possible consequences of migraine mutations affecting the central glutamate synapse. Increased Ca^2+^ influx caused by mutations in familial hemiplegic migraine subtype 1 (FHM1) encoding Cav2.1 channels enhance glutamate release from presynaptic terminals. Loss of Na^+^/K^+^ ATPase function, occurring in FHM2, also reduces astrocyte uptake of glutamate leading to increased levels of the neurotransmitter in the synaptic cleft. The mutations in FHM3 can reduce firing of inhibitory interneurons or potentiate presynaptic action potential generation. Mutations in FHM5/*SLC4A4* might inhibit glia-mediated acid secretion and thus free *N*-methyl-d-aspartate (NMDA) receptors from proton-mediated inhibition. Activity of EAAT1, the major glutamate transporter in the brain encoded by FHM6/*SLC1A3*, is directly affected by a mutation in its sequence and indirectly by a reported mutation in *rs1835740*, a variant located between *MTDH* and *PGCP* which might be up-regulated). The *LRP1* gene has a role in glutamate signalling and the consequence of a reported mutation in *rs11172113* might directly modulate NMDA-dependent calcium currents in vitro

On the basis of the aforementioned observations, it is tempting to hypothesise a unifying mechanism for FHM, able to explain at least the issues related to CSD. Mutant Ca_V_2.1 calcium channels might predict increased glutamate release in the cerebellar cortex, and accordingly, more easily induce, maintain, and propagate CSD [18]. FHM2 mutations predict reduced glial uptake of K^+^ and glutamate from the synaptic cleft whereas FHM3 mutations are expected to be associated with hyperexcitability of excitatory neurons. Thus, the predicted consequence of FHM1, FHM2, and FHM3 mutations is increased levels of glutamate and potassium in the synaptic cleft (see also Fig. 2). High glutamate and potassium conditions suggest an increased propensity for CSD which would provide a good explanation of the aura. It remains debatable, and largely unclear, whether this also results in a more readily activated trigemino-vascular system, and is therefore sufficient to explain the headache.

As the main clinical symptoms of headache and aura are similar in FHM and common migraine, it is thought that they may share a common pathophysiology [48]. Several studies have investigated (with conflicting results) the role of FHM1 and FHM2 loci/genes in the common forms of migraine since the early description of the CACNA1A gene in the late 90s. Variants in FHM3 are far less investigated. In short [49], these studies led to the conclusion that common variants in ion transport genes do not play a major role in susceptibility to common migraine. However, the possible role of rarer variants or variants with a smaller effect size cannot at present be predicted.

## Other forms of syndromic migraine

Excluding a major role for FHM genes in the aetiology of common forms of migraine does not mean that ion channels are not important to understanding of the pathogenesis of typical attacks. The same applies to other forms of “syndromic” migraine (see Table 2), all characterised by headache attacks of the migraine type. Clinical conditions such as cerebral autosomal dominant arteriopathy with subcortical infarcts and leukoencephalopathy (CADASIL) or mitochondrial DNA-related disorders, such as MELAS (mitochondrial encephalopathy with lactic acidosis and stroke-like episodes) may well shed light on important steps in the pathophysiology of aura or CSD but are less likely to account for a significant proportion of typical migraine attacks, even though headache pain is common among the plethora of neurological manifestations of both conditions. CADASIL is a brain microangiopathy due to mutations in the *NOTCH3* gene and it is characterised by stroke-like episodes, cognitive decline, MA (in about 35 % of cases), psychiatric disorders, and epilepsy [50]. Ischaemic attacks in CADASIL occurring during pregnancy and puerperium are more frequent in women aged over 30 years [51]. Additional symptoms include reversible acute encephalopathy [52], subclinical peripheral neuropathy, subclinical retinal vascular abnormalities [53], and occasionally acute myocardial infarction [54]. It remains possible that *NOTCH3* variants per se, or in combination with other functional modifications in an adjacent gene yet to be identified, are able to influence subtypes of migraine. Interestingly, it has been shown a positive allelic and genotypic association between a polymorphic variant in *NOTCH3* (namely, *rs 1043994*, c.684G>A) and MA in two independent population [55].

**Table 2:** A short list of syndromic clinical conditions presenting with migraine headache

Syndromic migraine	Gene (chromosome) involved	Migraine features
Familial hemiplegic migraine (FHM)	*CACNA1* (19p13); *ATP1A2* (1q23); *SCN1A* (2q24); others unknown	Attacks of hemiplegic aura
Mitochondrial encephalomyopathy, lactic-acidosis, stroke-like episodes (MELAS)	*MTTL1* (mtDNA)	Recurrent MA, focal neurological deficits, vomiting, convulsions
Cerebral autosomal dominant arteriopathy with subcortical infarcts and leukoencephalopathy (CADASIL)	*NOTCH3* (19q13.2)	MA/MO in 22–40 % of affected patients
Retinopathy, vascular, cerebral and renal involvement, Raynaud and migraine attacks (HERNS)	*TREX1* (3p21.3)	Migraine in most cases

*MA* migraine with aura, *MO* migraine without aura

MELAS is often associated with the mtDNA A-to-G transition at nucleotide 3243 and clinical features include seizures, hemiparesis, hemianopsia, cortical blindness, migraine and episodic vomiting [56]. In addition, systemic manifestations of MELAS, including cardiac, renal, endocrine, gastrointestinal, and endothelial abnormalities have been reported. Vascular system involvement has been highlighted in both conditions, and decreased oxidative brain metabolism has been shown to play a pivotal role in the pathogenesis of migraine [57].

## Linkage analyses and genome-wide association studies in common migraine

The involvement of three major genes in syndromic migraine is not paralleled by equally common aetiologies in common migraine, which suggests that other genetic approaches are needed to unveil the molecular basis of frequently occurring disorders such as MA and MO. Both linkage analyses and genome-wide association studies (GWAS) tend to detect alleles of medium–small effect size but high frequency. Linkage analyses investigate the co-segregation of diseases with genetic markers within family members. Although linkage methods proved to be extremely successful in monogenic Mendelian diseases, their power of detection is minimal when they are used to explore the genetic bases of complex traits and multifactorial diseases (such as migraine) not showing a simple Mendelian pattern of transmission (refer also to the detailed literature list cited in [58]). Most results prove to be “false” positive, fail to be replicated in larger cohorts, or are contradictory. Table 3 summarises several reproducible studies in both autosomes and the X-chromosome [58]. Despite sometimes being extremely rewarding (see the identification of the *TRESK* gene [59]), few linkage studies have been found to be replicable in independent investigations and their findings have pointed generally to broad genomic regions without clearly advancing our understanding of the genetics of typical MA or MO. The absence of clear biomarkers of disease status, the confounding role of spouses—spouses are generally sampled as healthy family controls but could themselves have contributed to the geno/phenotypes of their offspring—and the use of different diagnostic methods (direct ascertainment versus telephone interview) are able to explain only partly the poor reproducibility of these studies [9]. The use of more clearly defined individual features of migraine (‘‘endophenotypes’’) or analyses in isolated populations (see for reference the studies in Norfolk Island [60]) would result in a more powerful approach, as would the use of modern genetic technologies. One such approach is that of genome-wide data mining on automatic array platforms in which hundreds of thousands of single nucleotide polymorphic variants (SNPs) are simultaneously queried in large populations of migraineurs and non-migraineurs. GWAS have a high power to detect common variants of high or moderate effect size. For variants with smaller effect sizes (e.g. relative risk < 1.2), the power is greatly reduced, particularly for recessive loci. Excellent published works have employed GWAS to identify genetic markers specific for migraine (or its subtypes) compared with non-migraine headache. For further discussion of this topic, readers are referred to an outstanding review on GWAS power and significance in migraine, published in this journal earlier this year [9]. Even more recently, a GWAS study identified the first susceptibility loci specific for MO, thereby expanding our knowledge of this debilitating neurological disorder [61]. Several attracting markers have been pinpointed. Among these *rs1835740* seems to modulate glutamate homeostasis, *rs11172113* (in the *LRP1* gene, belonging to the lipoprotein receptor family) may also impact glutamate pathways through interaction with NMDA glutamate receptors (Fig. 2), whereas *rs10166942* (in *TRPM8*, encoding a cold and cold-induced burning pain sensor necessary for nociception) [9] implicates a pain-related pathway in migraine. Further work is required to confirm that the identified SNPs (such as *rs11172113* and *rs10166942* that have also been replicated in [61]) and adjacent genes are causally related and relevant to migraine. However, it is important to reiterate that GWAS studies should be conducted on large samples and ensure good clinical diagnosis and powerful technology and statistics.

**Table 3:** Loci identified in common forms of migraine and reproduced in independent studies (see reference [9] for details)

Chromosome locus	Phenotype	Methodology
1q22	MO	Genome-wide scan
3p24	MO	Genome-wide scan
4q21	MO	Genome-wide scan
4q24	MA	Genome-wide scan
5q21	Pulsating headache	Linkage analyses
10q22–q23	MO/MA	Linkage analyses
Xp22	MA	Linkage analyses
Xq24–q28	MA/MO	Regional microsatellite markers

*MA* migraine with aura, *MO* migraine without aura

Certainly, with powerful whole-genome assay technology rapidly becoming less expensive, the use of candidate gene association studies within the migraine scientific community seems superseded. That said, specific clinical conditions or variants of endophenotypes, or even the adoption of array-based platforms to assay multiple “attractive” or “hypothesis-drive” candidates in combination with rigorous meta-analyses, might still provide useful information on individual phenotypes. This might be true of variants in the glutamate receptor genes, or in hormonal or insulin receptors, or in the gene encoding the 5,10-methylenetetrahydrofolate reductase enzyme. For a more complete list, please refer to the conclusions of the study cited as Ref. [62].

## The outlook: next-generation migraine research

Technical advances in genomic sequencing are commonly referred as next-generation sequencing (NGS). These platforms are able to generate more sequence data and are substantially less expensive than the original “capillary” Sanger methods. Moreover, in the bid to account for structural variations, they can also handle more complex and smaller genomes, copy number variants, and SNPs [63]. Due to their cost-effectiveness and versatility, NGS approaches are poised to emerge as a dominant genomics technology in patient-oriented research. Specifically, there is considerable interest in employing NGS platforms for targeted sequencing of specific candidate genes and sequencing of SNPs identified through GWAS. As an example, targeted genomic enrichment approaches with NGS enable deep sequencing of any complex genomic region of interest, and may be a straightforward means of detecting causal variants in common diseases, capable of contributing to understanding of their pathogenesis. With the falling cost of NGS technology, sequencing of the entire human exome in large numbers of individuals is now feasible and promising [64].

We anticipate that in the near future, with the costs of targeted multiplex amplicon enrichment also falling, NGS applications will flourish the field of migraine genetics. It is to be hoped, in particular, that this more intensive application might bring particular advantages in terms of efforts to improve the individual response to drug treatment, which is the final goal of molecular genetic studies in migraine. Advances have actually already been made in the field using more traditional “capillary” sequencing approaches. This is true in the case of chronic migraine, a clinical [65] and pathophysiological [66] challenge for headache researchers. For instance, the serotonin pathway was intensely investigated in patients with chronic migraine associated with medication overuse headache (MOH) but none of the SNPs analysed in genes encoding serotonin transporters and receptors 1A, 1B, 2A and 6 [67, 68] could be firmly linked to the development of MOH. Conversely, genetic polymorphisms in MAO-A and CYP1A2 were found to be over-represented in MOH patients as compared to sporadic migraineurs [69]. A polymorphism of the brain-derived neurotrophic factor gene and a polymorphism of the *WFS1* gene (related to several psychiatric disorders) were also found to be related to higher monthly analgesic intake [70, 71]. Pending replication studies and meta-analyses, we cannot draw solid conclusions about the existence of a genetic determinant predisposing to chronicity [68]. Importantly, given the high rate of patients not responding to common prophylactic and symptomatic therapies, the use of modern technologies (i.e. NGS) might fit well into recent pharmacogenomic strategies [72, 73] that are addressing the issue of how genetic determinants influence drug response. Nonetheless, the complexity of this field is such that even higher predictive power and careful clinical investigations will be needed before applications can be translated into clinical practice. The study of genetic factors of individual response (pharmacogenomics) in terms of efficacy or adverse events to prophylactic treatments [74] might be relevant in this regard. It is, indeed, surprising that most studies focus only on symptomatic therapies with only a handful [72, 75] being devoted to the pharmacogenomics of effective preventive management of migraine. NGS has the potential to fill the knowledge gap in the pharmacogenomics of migraine, generating a positive fallout on daily clinical practice.

In conclusion, we expect to see an increase in understanding of the molecular genetics of migraine, even though the disease itself does not really seem to be a “canonical” genetic condition. Novel molecular approaches should increase our chances of finding answers to still open questions, such as “how migraine attacks start” or “why headache occurs in MA and MO”. Wider use of NGS applications will not only scan the individual genome, with a view to future personalised therapies, but also provide information on epigenetic mechanisms, that is, modifications in gene expression such as gene methylation that are heritable but are not encoded in the DNA sequence. Although these modifications do not impact on the nucleotide sequence of the DNA, they have the potential to modulate gene expression and influence molecular pathways. New research directions are likely to focus on understanding the various factors (not only genetic) that are well balanced in healthy subjects but disturbed in migraineurs.
